# Comparison of Lifestyle in Women With Polycystic Ovary Syndrome and Healthy Women

**DOI:** 10.5539/gjhs.v7n1p228

**Published:** 2014-08-31

**Authors:** Sedigheh Sedighi, Sedigheh Amir Ali Akbari, Maryam Afrakhteh, Taraneh Esteki, Hamid Alavi Majd, Zohreh Mahmoodi

**Affiliations:** 1 Department of Midwifery, Nursing and Midwifery School, Shahid Beheshti University of Medical Sciences, International Branch, Tehran, Iran; 2 Departments of Midwifery, Nursing and Midwifery School, Shahid Beheshti University of Medical Sciences, Tehran, Iran; 3 Departments of Obstetrics and Gynecology, Shahid Beheshti University of Medical Sciences, Tehran, Iran; 4 Department of Basic Sciences, Nursing and Midwifery School, Shahid Beheshti University of Medical Sciences, Tehran, Iran; 5 Departments of Biostatistics, Paramedicine School, Shahid Beheshti University of Medical Sciences, Tehran, Iran; 6 Departments of Midwifery, Nursing and Midwifery School, Alborz University of Medical Sciences, Karaj, Iran

**Keywords:** polycystic ovary syndrome, lifestyle, unhealthy behaviors, IPAQ, diet and PCOS, physical activity and PCOS

## Abstract

Given the high prevalence of polycystic ovary syndrome (PCOS), and that a lifestyle is recognized effective in development of many diseases, this study aimed to compare lifestyle of women with PCOS and healthy women. Nor are there sufficient studies on the difference between lifestyle of these people with that of healthy people. Furthermore, studies show that changes in lifestyle improve this disease. This descriptive-comparative study was conducted on 65 women with PCOS and 65 healthy women of 18 to 45 years old who presented to hospitals affiliated to Shahid Beheshti University of Medical Sciences in 2013. The subjects were selected using multi stage random sampling method. The data were collected using questionnaires for diet, International Physical Activity Questionnaire, and unhealthy behaviors and were analyzed in SPSS v. 17, using descriptive statistics, Man-Whitney, independent t, Chi-square and logistic regression tests. The results showed there was a significant relationship between PCOS and inappropriate diet (p=0.009), low physical activity (p=0.009), but no relationship was observed between PCOS and unhealthy behaviors. Given the results obtained, training and awareness raising is necessary for women and girls especially about appropriate diet and regular physical activity.

## 1. Introduction

Polycystic ovary syndrome (PCOS) is the most prevalent endocrine disorder in women at reproductive ages, and the most common cause of hyperandrogenism and hirsutism. The prevalence of PCOS varies from 2.2% to 26% in different countries and has been reported as 7.1% based on National Institutes of Health (NIH) criteria, as 11.7% based on Androgen Excess Society (AES) criteria, and as 14.6% based on Rotterdam criteria in Iran (Ramezani Tehrani et al., 2011). It is estimated that 105 million people suffer this syndrome among 15- to 49-year-old women worldwide (Nasiri Amiri, Ramezani Tehrani, Simbar, & Mohammadpour Thamtan, 2013). This syndrome incurs heavy costs on healthcare systems in different countries in that in the U.S. in 2004, 4.36 billion USD was spent only to treat PCOS and its complications. More than 40% of this cost was spent on infertility and menstrual period impairments and another 40% was spent on treatment and diabetes control (Harisson, Lombard, Moran, & Teede, 2010). Women with PCOS are at risk of fertility problems (mense disorders, failure to ovulate, late menopause, endometrial cancer and infertility), metabolic problems (insulin resistance, diabetes type 2, dyslipidemia, hypertension, and cardiovascular diseases), physical problems (central obesity, acnea, hirsutism, hair loss and baldness), and psychological problems (depression, stress and anxiety) (Bozdag & Yildiz, 2013; Balakrishnan, 2013; Lass, Kleber, Winkel, Wunsch, & Reinehr, 2011). The familial incidence of this syndrome has been well established and its prevalence and symptoms vary in different ethnicities (Allahbadia & Merchant, 2011). The main cause of this syndrome is unknown, but there is evidence of several genes involved, which work in appropriate environmental circumstances, especially nutritional factors (V. Tomic & J. Tomic, 2012). Some studies show evidence of dominant x-linked inheritance (Badawy & Elnashar, 2011). World Health Organization study on health behaviors of 35 countries showed that 60% of people’s quality of life and health depends on their lifestyle and personal behaviors. One of the objectives of WHO for 2020 is to promote healthy lifestyle, reduce health risk factors including inappropriate physical activity, bad diet, defective interpersonal relationships, alcohol use, and substance use (Curi et al., 2012). Correcting lifestyle, first introduced by the Austrian psychoanalyst Alfred Adler, is an important concept that is mostly used to express people’s lifestyle and covers different dimensions including nutrition, exercise, self-care, cigarette smoking, alcohol and substance use, social support, and stress control (Kamali Fard, Alizadeh, Sehati, & Gojazadeh, 2010). Lifestyle is closely related to physical and mental health of people, and is effective in onset or development of many diseases. There is a lot of evidence that shows lifestyle, diet and obesity in childhood and adulthood are risk factors for chronic diseases (Shahedur, Anowar, Abdus, & Shahjahan, 2012). Although obesity has not been mentioned as a diagnostic criterion, it is a major factor in incidence and intensity of the disease. It is estimated that approximately 40%-60% of these people are obese. Obesity aggravates the clinical presentation of the disease in terms of both fertility and metabolism. Insulin resistance is quite common among obese people such that 70%-80% of the obese people suffer from it. Abnormal glucose metabolism significantly improves with weight loss. Weight loss can reduce hyperandrogenism and improve ovulation. In obese insulin resistant women, limited calorie intake can reduce weight and insulin resistance (Hoeger, 2012). Exercise regardless of weight loss reduces insulin resistance, but there are few data on the effect of exercise on major presentations of the disease (Novak, Berek, Hillard, & Adashi, 2012). The association between bad nutritional habits and risk of the disease has not been studied well, and the existing data are scarce and contradictory (Altieri et al., 2013). Several studies have tried to prove the role of exercise on treating the obese women with PCOS. Exercise has been recommended, but the type, duration and frequency of exercising are controversial (Badawy & Elnashar, 2011). Healthy adolescents have more physical activity than their peers with PCOS (Eleftheriadou et al., 2012). Regular physical activity, as an important health promoting behavior can prevent or delay different types of chronic diseases and early death. Several studies have proved that smoking can reduce estrogen and increase androgen in women in reproductive ages (Pau, Keefe, Corrine, & Welt, 2013). This disease involves several physical problems and several studies have shown that lifestyle changes including more physical activity, changes in nutritional habits and diet and quitting smoking can improve obesity, metabolic and hormonal status in these people. This syndrome is highly prevalent and has several short term and long term complications. There are no known causes and cure for this disease, nor are there sufficient studies on the difference between lifestyle of these people with that of healthy people. Furthermore, studies show that changes in lifestyle improve this disease, and that lifestyle affects the onset or progress of chronic diseases and that all aspects of lifestyle are closely related to one another. Considering all the above-mentioned points, the present study was conducted because the prevalence of lifestyle-related diseases is increasing and there are not enough studies in this regard. This study was conducted to compare baseline physical activity, feeding method, and unhealthy behaviors in patients (who did not undergo any treatment, food diet, or regular exercising program) with those in normal people in order to identify the differences, if any, in normal lifestyle of these two groups and be useful for further studies on causes of these differences and their effect on the incidence or exacerbation of the disease.

## 2. Materials and Methods

### 2.1 Study Design

This descriptive-comparative study was conducted on 65 women with PCOS and 65 healthy women presenting to hospitals affiliated to Shahid Beheshti University of Medical Sciences in 2013. Inclusion criteria are: age between 18 and 45 years old, Iranian and Persian speaking, ability to read and write, no known medical conditions according to self-report or using patients’ records including diabetes, hypertension, thyroid disorders, hyperprolactinemia, Cushing’s syndrome, adrenal hyperplasia, no antidepressant medication, no hormone therapy, no oral contraceptive, no glucocorticoids, no anti-obesity medication, no special diet, and not being a professional athlete. Women were selected based on Rotterdam criteria (at least two out of three criteria) including amenorrhea, oligomenorrhea, clinical or lab evidence of hyperandrogenism including hirsutism, alopecia, acne or increased serum levels of androgens, ultrasound evidence of PCOS including more than 12 follicles in each ovary with a diameter of 2-9 mm or increased volume of ovary to more than 10 cm^3^, and confirmation of the gynecologist in the center. Healthy women were selected from women visiting the same hospitals, but without signs of the disease according to Rotterdam criteria. Sample size was determined using the below formula with CI 95% as 65 people in each group.

*n= 2 (Zα + Zβ) ^2^σ^2^/ (μ_1_-μ_2_)^2^* (1)

In this equation, type I error, test power, and effect size in estimation of differences in lifestyle were determined in both groups as 5%, 80%, and 0.5, respectively.

### 2.2 Measurement Instrument

Data collection tools were demographics, diet, IPAQ^1^ and unhealthy behavior questionnaires, tape measure and scale. Demographics questionnaire included age, height, weight, BMI, education level, marital status, if married: husband’s education, monthly income, menstruation history such as menarche, interval between menses, obstetrics history like parity, number of miscarriages, number of preterm births, and use of assisted fertility methods. The researcher-made diet questionnaire comprised 28 items with 0 to 112 points, where higher score showed more appropriate diet. There were 16 items for positive diet and 12 items for negative diet. Total score was measured by considering from 4 points for daily to 0 for never regarding the positive items and vice versa for the negative items. The total score was converted to 0 to 100 for statistical analysis, and 0-33, 34-66 and 67-100 were considered for inappropriate, quite appropriate and appropriate diet, respectively. Content validity was used to validate the questionnaire. Its reliability was measured as 0.80 using Cronbach’s alpha. IPAQ was developed in 1998 in Geneva by WHO and CDC for age groups of 15-69. It has 27 items that report physical activity based on MET-min.week. One MET equals the amount of energy consumed in one minute of rest. IPAQ classifies people based on MET into 3 groups of low activity (<600 MET), average activity (600-3000 MET) and high activity (>3000 MET). This questionnaire is standard and its validity has been confirmed using content validity in previous studies (Dinger, Behrens, & Han, 2006; Hazavehei, Asadi, Hassanzadeh, & Shekarchizadeh, 2008). Its reliability was reported as 0.80 in 12 countries by Craig et al. in 2003 (Craig et al., 2003). Unhealthy behavior questionnaire was researcher-made with 8 items and 22 points. The scores were converted to 0-100, where 0-33, 34-66 and 67-100 respectively showed the lowest, moderate and the highest high risk behaviors. Content validity was used to validate this questionnaire, and retest was used to determine its reliability. A non-flexible metal tape measure (Laika, Italy) and a scale (Secca, Germany) were used to measure height and weight, respectively. The scale was validated using a standard 1-kg weight, and calibrated with the same weight after every 10 times of weighing, according to manufacturer’s recommendation. The reliability of the tape measure was measured using a standard non-flexible tape measure that was not affected by climatic conditions. The data were analyzed in the SPSS v 17 software and an alpha level of 0.05 for all statistical tests. Independent t test was used to compare pairs of data in groups if the data had normal distribution, Man-Whitney test was used if the data did not have normal distribution, and Chi-square test was used for qualitative data. Finally, logistic regression was used for the whole set of data.

### 2.3 Data Collection and Interventions

After obtaining the permission from authorities of Shahid Beheshti University of Medical Sciences and the heads of hospitals, the women with PCOS and healthy women were identified using multi stage random sampling, and were invited to participate with respect to ethical considerations. After they were briefed about the objectives and methods of the study, and were reassured about the confidentiality of the information, they signed written consent form and were enrolled. The questionnaires were filled out by the researcher in about 10 minutes. Sampling continued until enough samples were selected. The subjects were matched for age, and body mass index (BMI).

## 3. Results

In this study, 65 women with PCOS and 65 healthy women participated. The mean age was 28.85±6.525 and 29.57±7.794 years in the PCOS group and the healthy groups, respectively. The mean BMI was 24.02±3.48 and 23.47±3.281 kg.m^2^ in the PCOS and the healthy groups, respectively. There was no significant difference between the two groups. There was no difference between the two groups in terms of subjects’ education, their husbands’ education, subjects’ occupation, their husbands’ occupation, monthly income and marital status. (Table 1 demonstrates demographics of the subjects).

**Table 1:** The absolute and relative frequency distribution of demographics in the two groups

Variable	PCOS (65) number	Non-PCOS)65) number	P- value
Education	37 high school	29 high school	0.260*
Husbands’ education	15 high school	12 high school	0.410*
Occupation	44 housewife	55 housewife	0.170**
Husbands’’- occupation	20 self- employed	19 self- employed	0.841**
Monthly income <600 thousand Rials	28	25	0.775*
Marital status	35 married	34 married	0.860**

* U Man-Whitney

** Chi-square.

There was no significant difference between the two groups in terms of infertility, history of assisted fertility methods, age at menarche (p=0.172), and the number of miscarriages (p=0.142). Nonetheless, there was a significant difference between the two groups in terms of parity (p=0.004) and the number of preterm births (p=0.049).

Table 2 demonstrates the comparison of the two groups in terms of diet, physical activity and unhealthy behaviors. there was a significant difference between the two groups in terms of diet and physical activity (p<0.001). The mean score of unhealthy behaviors was 6.43 and 5.94 in the PCOS group and the healthy group, respectively, but the difference was not significant (p=0.7).

**Table 2:** Comparison of mean scores of different dimensions of lifestyle in the two groups

Variable	PCOS (65)	Non-PCOS)65)	P- value
Diet	58.34±7.55	68.50 ±8.87	<0.001*
Physical activity (MET)	809.85±629.19	1916.8±1708.88	<0.001**
Unhealthy behaviors	6.43±11.19	5.94±11.36	0.7*

* t-test

** U Man-Whitney.

As can be seen in Table 3, bivariate logistic regression model shows that diet, and physical activity significantly affect affliction with PCOS.

**Table 3:** Calculating regression coefficients of lifestyle and demographics in the two groups*

Variable	OR	P- value	CI (95%)
Age (years)	0.875	0.109	0.743-1.030
BMI (kg.m2)	0.971	0.827	0.742-1.270
Education	0.895	0.810	0.364-2.203
Occupation	1.188	0.660	0.551-2.562
Age at menarche (years)	0.643	0.050	0.413-0.999
Menarche interval (day)	0.243	0.023	0.072-0.824
Diet	0.925	0.009	1.019-1.147
Physical activity(MET)	0.930	0.009	1.019-1.135
Unhealthy behaviors	0.992	0.837	0.963-1.054

* Based on bivariate logistic regression model.

## 4. Discussion

The analysis of the data showed a significant relationship between PCOS and inappropriate diet (p=0.009), low physical activity (p=0.009). In recent years, researchers have attended to factors involved in incidence or severity of PCOS. Nutritional habits are one dimension of lifestyle which affects people’s physical health (Shahedur et al., 2012). Based on the results of our study, the two groups were significantly different in terms of the mean consumption of different food groups in food pyramid.

Few studies have been conducted on lifestyle and nutritional habits of people with PCOS (Barr, Hart, Reeves, Sharp, & Jeanes, 2011). Appropriate diet is an important factor in initiating and maintaining normal function of puberty and fertility (Altieri et al., 2013). Nowadays, correcting lifestyle through adjusting diet and exercising aim to normalize androgen levels and set ovulation is considered as the first line of treatment for these patients (Novak et al., 2012). Some studies have reported a strong relationship between increased risk of infertility due to failure to ovulate and high consumption of animal proteins, complete carbohydrates, foods with high glycemic index, low fat dairy and sodas (Altieri et al., 2013). Insulin resistance causes hyperandrogenemia and disrupts normal ovary function, but dairy products reduce insulin resistance, and low fat dairy is related to hyperandrogenemia (Pourghassem Gargari, Houjeghani, Mahboob, Farzadi, & Safaeian, 2011). Pourghassem Gargari et al. (2011) assessed the pattern of receiving nutrients in women with PCOS and healthy women, and reported that mean intake of energy, carbohydrates, fat and protein was higher in control group. Furthermore, the frequency of consuming milk and dairy, fruits and nuts was less in women with PCOS (p<0.05). The results of this study were inconsistent with those of the present study in terms of intake of carbohydrate, fat and protein, but consistent in terms of consuming milk, dairy, fruits and nuts. Eman et al. (2012) reported higher calorie intake (p=0.001) and fat intake (p=0.019) in women with PCOS, which is in line with the present study. Altieri et al. (2013) studied food habits of obese women with PCOS and its relationship with hormones and metabolism, and observed that the mean consumption of cookies (p=0.013), cheese (p=0.049) and greasy foods (p=0.048) was higher in the PCOS group than in the control. The results of this study are consistent with ours regarding consuming cookies and greasy food, and inconsistent regarding consuming cheese. Given the fact that in most studies, people’s hormonal, metabolic and clinical status improved after changing their diet, and the role of obesity and inappropriate nutrition has been recognized effective in this disease, it is necessary to provide them with consultation and educational services regarding appropriate nutrition. We observed that women with PCOS had less activity than healthy women regarding average and high intensity activity. Nowadays, correcting lifestyle through diet and exercising which normalized androgen levels and improves ovulation is the first line of treatment in these patients (Hoeger, 2006). Exercises that involve larger muscles can reduce insulin resistance and comprise an important part of non-medical treatment (Moran, Brink worth, & Norman, 2008). Exercise and physical activity reduces body fat, which is the storage for estrogens and steroid hormones (Alijani & Hiatgheibi, 2002). Although increased physical activity causes better glucose metabolism, increases sensitivity of cells to insulin and reduces abdominal fat regardless of weight loss, studies show that physical activity of people with PCOS is really low (Barr et al., 2011). The results of the study by Eleftheriadou et al. (2012), titled ‘exercise and low activity in people with PCOS’ showed that girls with PCOS are less inclined to do physical activity (p<0.001), and when they attended, its frequency and intensity were lower than those of the control group. These results were true for thin people with PCOS as compared with the control. The results of this study are in line with ours. However, the number of sitting hours was not significantly different in the two groups in this study, which is against our findings. Moran et al. (2013) conducted a study on the effect of diet, physical activity and sedentary lifestyle on BMI in women with PCOS and healthy women for 13 years (1996-2009) in Australian women born between 1973 and 1978. They did not report a significant difference between the two groups in terms of physical activity, which is inconsistent with our results. They reported a significant difference between the sitting hours of the two groups, which is in line with our findings. Haqq et al. (2014) conducted a meta-analysis titled lifestyle interventions and endocrine presentation in PCOS, and concluded that physical activity improved SHBG, FSH, testosterone and androstenedione in those with PCOS. Our results in this regard show that low physical activity was low in both groups, which is in line with previous studies. Given the role of exercising in preventing the incidence or progress of many diseases and its positive effect on improving hormonal status and insulin resistance in these people, it is recommended to provide them with consultation and educational services.

There was no significant relationship between the mean score of unhealthy behaviors between the two groups. Several studies have proved that smoking reduces estrogen levels and increase androgen levels in women at reproductive age. Therefore, smoking increases metabolic syndrome and hyperandrogenism in women with PCOS (Pau et al., 2013). Glintborg et al. (2012) studied 650 white women with PCOS that comprised 390 smoking and 260 non-smoking women. Smoking was found related to increased adrenal response, reduced prolactin level, and aggravated fat profile, but multiple regression showed that PCOS was more prevalent in non-smoking women. In our study, we did not observe a high level of unhealthy behavior and average unhealthy behavior was observed only in 4 people in the PCOS group and 3 people in the healthy group. It seems that sample size was not enough for this test. The researcher recommends a more extensive study in this regard. These data are referring to Iran population because among the items studying there are also nutrition and lifestyle that vary from country to country, and this was a limitation of this study.

## 5. Conclusion

The results showed that there is a difference between lifestyle of people with PCOS and those without in several domains. The need to attend to nutrition and physical activity in women and girls is clear.
